# Lecanemab Slows Tau PET Accumulation

**DOI:** 10.1002/alz.091956

**Published:** 2025-01-09

**Authors:** Arnaud Charil, Youfang Cao, Brian A. Willis, Steven Hersch, Michael C. Irizarry, Larisa Reyderman

**Affiliations:** ^1^ Eisai Inc., Nutley, NJ USA; ^2^ Deep Human Biology Learning (DHBL), Eisai Inc., Nutley, NJ USA

## Abstract

**Background:**

Lecanemab is an approved anti‐amyloid monoclonal antibody that binds with highest affinity to soluble Aβ protofibrils, which are more toxic than monomers or insoluble fibrils/plaque. In clinical studies, biweekly lecanemab treatment demonstrated a slowing of decline in clinical (global, cognitive, functional, and quality of life) outcomes, and reduction in brain amyloid in early Alzheimer’s disease (AD). Herein, we describe the impact of lecanemab treatment on tau PET.

**Method:**

Changes over time in tau PET SUVR ([18F]MK6240) were compared between placebo and lecanemab‐treated groups. Associations between baseline and change from baseline (CFB) in tau PET SUVR across brain regions were investigated. A quantitative systems pharmacology (QSP) model was developed to describe the dynamics of medial temporal (MTL) tau PET based on AD pathophysiology with three interlinked modules: Aβ pathway, tau pathway, and cognitive function. MTL tau PET data from the lecanemab Clarity AD study and other biomarkers from Clarity AD and lecanemab Study 201, and Alzheimer’s Disease Neuroimaging Initiative (ADNI), were used to inform and validate the model. QSP model‐based simulations were conducted to investigate the effect of lecanemab treatment on tau pathology.

**Result:**

Higher baseline tau levels were associated with higher tau accumulation across brain regions with disease progression. Lecanemab treatment significantly slowed accumulation of tau in temporal regions relative to placebo and disrupted association between baseline tau and CFB in tau across brain regions. QSP simulations predicted MTL tau PET accumulates 0.064 SUVR/year in placebo, and 0.092 SUVR/year in high baseline tau group (tau PET SUVR>1.5). QSP simulations predicted that lecanemab treatment halted tau PET accumulation in MTL region over 18 months of treatment and maintained suppression with continued biweekly lecanemab regardless of baseline tau levels.

**Conclusion:**

Lecanemab treatment demonstrated an impact on tau accumulation across brain regions. QSP model adequately described MTL tau PET for placebo and lecanemab across 30 months of Clarity AD data. QSP simulations suggest that lecanemab halts tau PET accumulation by clearing major tau pathology drivers of amyloid protofibril and plaque, slowing down long‐term cognitive decline. Taken together, tau PET data and QSP modeling support additional benefits of continued lecanemab treatment.

